# CD271^+^ Osteosarcoma Cells Display Stem-Like Properties

**DOI:** 10.1371/journal.pone.0098549

**Published:** 2014-06-03

**Authors:** Jiguang Tian, Xin Li, Meng Si, Ting Liu, Jianmin Li

**Affiliations:** 1 Department of Orthopedics, Qilu Hospital, Shandong University, Shandong, China; 2 Department of Obstetrics and Gynecology, Qilu Hospital, Shandong University, Shandong, China; Instituto Butantan, Brazil

## Abstract

Cancer stem cell (CSC) theory has been proposed and verified in many cancers. The existence of osteosarcoma CSCs has been confirmed for many years and multiple surface markers have been employed to identify them. In this study, we identified CD271^+^ subpopulation of osteosarcoma displaying stem-like properties. CD271, known as the neural crest nerve growth factor receptor, is the marker of bone marrow mesenchymal stem cells (MSCs) and human melanoma-initiating cells. We discovered that CD271 was expressed differentially in diverse types of human osteosarcoma and stabilized cell lines. CD271^+^ osteosarcoma cells displayed most of the properties of CSC, such as self-renewal, differentiation, drug resistance and tumorigenicity in vivo. Nanog, Oct3/4, STAT3, DNA-PKcs, Bcl-2 and ABCG2 were more expressed in CD271^+^ cells compared with CD271^−^ cells. Our study supported the osteosarcoma CSC hypothesis and, to a certain extent, revealed one of the possible mechanisms involved in maintaining CSCs properties.

## Introduction

Osteosarcoma is the most common primary malignant bone tumor in children and adolescents. Despite the intensified chemotherapy and aggressive surgery, the survival rates of osteosarcoma patients have remained at 50%–80% since 1970s [1], [2], [3]. Increasing evidences have supported the hypothesis that a small cell subpopulation displaying stem-like properties is responsible for cancer relapse and metastasis [4]. These cell subsets are called cancer stem cells (CSCs) or tumor initiating cells (TICs).

According to CSC theory, the bulk of tumor is comprised of heterogeneous cell population. CSCs are at the top of hierarchy. By symmetrical and asymmetrical division, the rare CSCs are capable of self-renewal and generating the rest of the growing tumor cells. Unlike normal stem cells, CSCs are out of control in proliferation and maintaining genomic integrity [5].

CSCs have been identified in many types of cancers, such as leukemia, breast tumor, brain tumor, prostate tumor and melanoma [6], [7], [8], [9], [10]. CSCs are identified mainly based on detection of molecule markers, intrinsic cellular properties and Functional characterization [11].

Since stem-like cells in bone sarcoma were firstly detected by Gibbs [12], multiple markers have been employed to identify CSCs of osteosarcoma, such as CD133 [13], CD117/Stro-1 [14], CBX3/ABCA5 [15]. CSCs with these marks shared similar stem–like properties, such as self-renewal, differentiation, drug resistance, tumorigenicity and multi-potency. Although osteosarcoma CSCs account for only few percentages of cells, they have advantages of survival, proliferation and oncogenicity compared with the rest.

CD271, known as one of the cell-surface markers of bone marrow mesenchymal stromal/stem cell (MSC) [16], [17], was recently reported being expressed in human melanoma-initiating cells [18]. The purpose of our study was to determine whether CD271^+^ osteosarcoma cells display stem-like properties. We have investigated the abilities of self-renewal, differentiation, drug resistance and tumorigenicity of CD271^+^ cells and then studied the possible mechanisms involved in maintaining these properties. Our study may be helpful in the development of targeted therapies in the future.

## Results

### CD271 Expression in human biopsy specimens and cell lines

**Figure 1:**
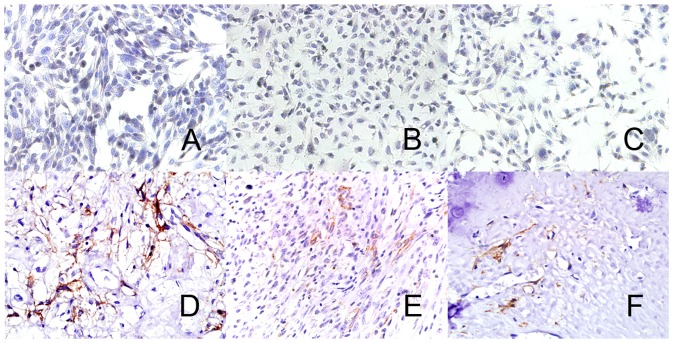
Human steosarcoma tissue and cell lines expressed CD271. Immunocytochemical staining of CD271 in osteosarcoma cell lines SAOS2(**A**), U2OS(**B**), MNNG/HOS(**C**). Immunohistochemical staining of CD271 in biopsy of different type of osteosacoma, osteoblastic(**D**), fibroblastic(**E**) and chondroblastic(**F**). Few percentages of cells displayed strong to medium positive expression of CD271 with a plasma membrane pattern. Magnification 400×.

**Figure 2:**
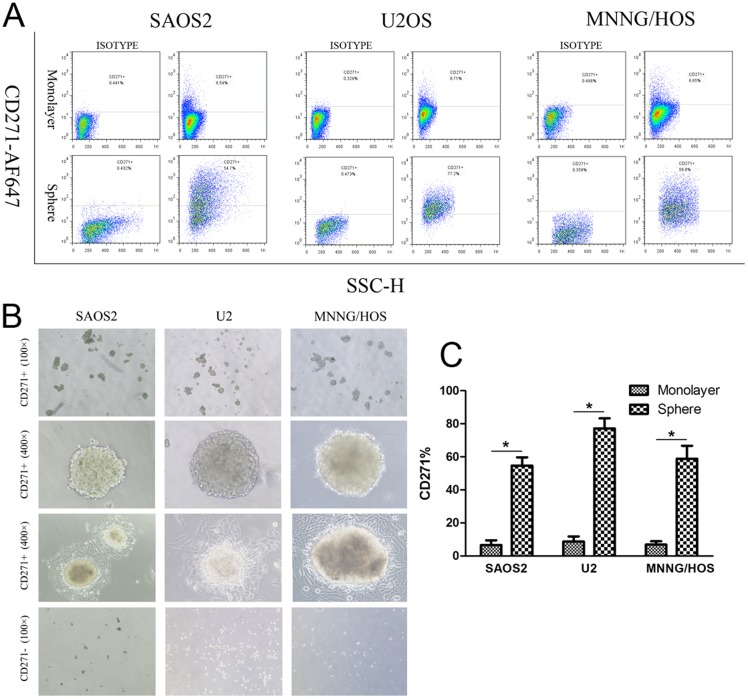
Sarcospheres had higher CD271 expression. (**A**, **C**) Sphere cells (A, bottom panel) had higher CD271 expression compared with monolayer cells(A bottom panel). (**B**)Spheres formation assay. CD271^+^ cells formed sarcospheres in anchorage-independent, serum-starved conditions (top and second panel). The sarcospheres detached into monolayers in normal condition (third panel). CD271^−^ cells hardly formed any sphere in anchorage-independent, serum-starved conditions (bottom panel). *P<0.01.

We found that CD271 was expressed in the tissue specimens, representative images of immunostaining for CD271 showed a plasma membrane pattern (Figure 1). The CD271 expression was varied in osteoblastic, chondroblastic and fibroblastic osteosarcoma (ranged from 0 to 29%). CD271 was also expressed in a small portion of cells in osteosarcoma cell lines, SAOS2(6.21±0.46%), U2OS(8.73±1.01%), MNNG/HOS(6.52±0.98%)(Figure 2A, up panel). These data indicated that CD271^+^ osteosarcoma cells maybe a new subpopulation with special properties distinguished from the rest.

### CD271^+^ cells had the abilities of self-renewal and differentiation

Increasing evidences have supported that sarcospheres obviously display stem-like properties [19], [20]. We detected the CD271 expression in sarcospheres of osteosarcoma cell lines SAOS2, U2OS, MNNG/HOS. Sarcospheres were cultured in anchorage-independent, serum-starved conditions, then dissociated and detected by flow cytometry (FCM). As a result, the elevated proportion of CD271^+^ cells was detected in sarcospheres of all three cell lines (Figure 2A, bottom panel).

In order to investigate the self-renewal and differentiation abilities, CD271^+^ cells were enriched by magnetic activated cell sorting (MACS), then cultured in anchorage-independent, serum-starved conditions. After 7–9 days, sarcospheres was formed in CD271^+^ cultures but not in CD271^−^ cultures. (Figure 2B). Next, sarcospheres were dissociated and cultured in anchorage-independent, serum-starved conditions repeatedly and the secondary spheres were formed in 6–8 days. Thereafter, sarcospheres were collected and cultured in adherent, serum-containing conditions. In 9–12 days, sarcospheres of three cell lines expanded into monolayers cells, and the CD271 expression recovered to the normal level of primary cell lines.

**Figure 3:**
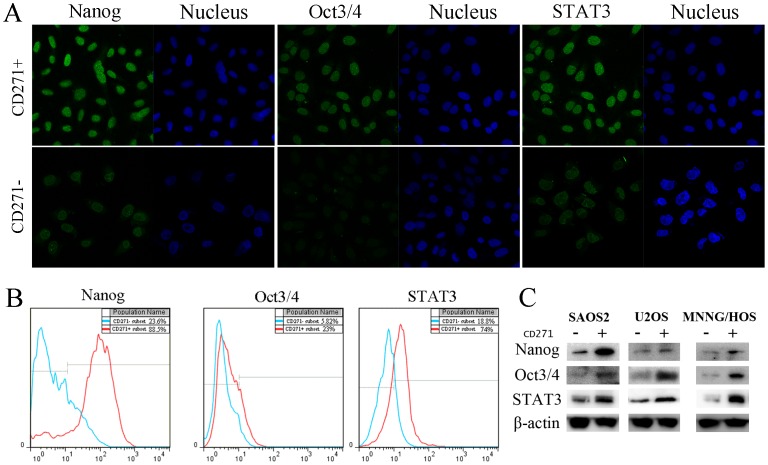
Expression of ES marker genes in CD271^+^/CD271^−^ cells. (**A**,**B**) Immunofluorescence and flow cytometry analysis demonstrated that Nanog, Oct3/4 and STAT3 are located in nucleus and expressed highly in CD271^+^ SAOS2 cell lines. (**C**)Western blotting analysis manifested higher expression of these proteins in CD271^+^ cells than in CD271^−^ cells of SAOS2, U2 and MNNG/HOS cell lines.

Two studies of murine osteosarcoma have confirmed that three transcription factors: Nanog, Oct3/4 and STAT3, are involved in maintaining self-renewal and pluripotency of ES cells [21], [22]. We assessed the expression of these proteins by performing western blot, immunofluorescence and flow cytometry in CD271^+^/CD271^−^ osteosarcoma cells. As shown in Figure 3, Nanog, Oct3/4 and STAT3 which are located in the nucleus of all three cell lines exhibited higher expression in CD271^+^ cells than in CD271^−^ cells.

### CD271^+^ cells were more resistant to DDP compared with CD271^−^ cells

**Figure 4:**
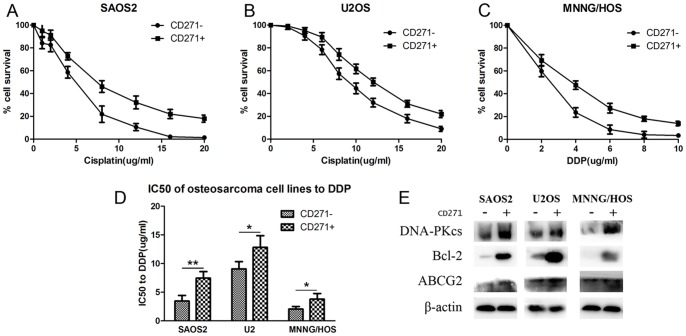
CD271^+^ cells were more resistant to DDP. (**A-C**) SAOS2, U2OS and MNNG/HOS cell lines were treated with DDP of different concentration for 24 h and then with drug free medium for another 24 h. Cell viability was tested by MTT assay. (**D**)IC50 to DDP was evaluated. CD271^+^ cells showed higher IC50 compared with CD271^−^ cells in SAOS2(7.46±1.13 ug/ml vs 3.45±0.97 ug/ml), U2OS(12.82±2.04 ug/ml vs 9.06±1.28 ug/ml), MNNG/HOS(3.4±0.97 ug/ml vs 2.06±0.44 ug/ml). (**E**)Western blotting analysis manifested higher expression of DNA-PKcs, Bcl-2 and ABCG2 in CD271^+^ cells. *P<0.05, **P<0.01.

Another property of CSCs is drug resistance which led to failure of chemotherapy and recurrence of tumor. We treated CD271^+^ and CD271^−^ cells with DDP of different concentration for 24 hours. As a result, CD271^+^ cells showed more resistance to DDP with higher IC50 values in all three cell lines(7.46±1.13 ug/ml vs 3.45±0.97 ug/ml in SAOS2, 12.82±2.04 ug/ml vs 9.06±1.28 ug/ml in U2OS, 3.4±0.97 ug/ml vs 2.06±0.44 ug/ml in MNNG/HOS, Figure 4, A-D). Next, the key protein, catalytic subunit of DNA-dependent protein kinase (DNA-PKcs), Bcl-2 and ABCG2, involved in DNA repair and anti-apoptosis was investigated by western blotting. As a result, in all three cell lines, DNA-PKcs, Bcl-2 and ABCG2 were more expressed in CD271^+^ cells than in CD271^−^ cells (Figure 4E).

### Cell proliferation and cycle analyses

**Figure 5:**
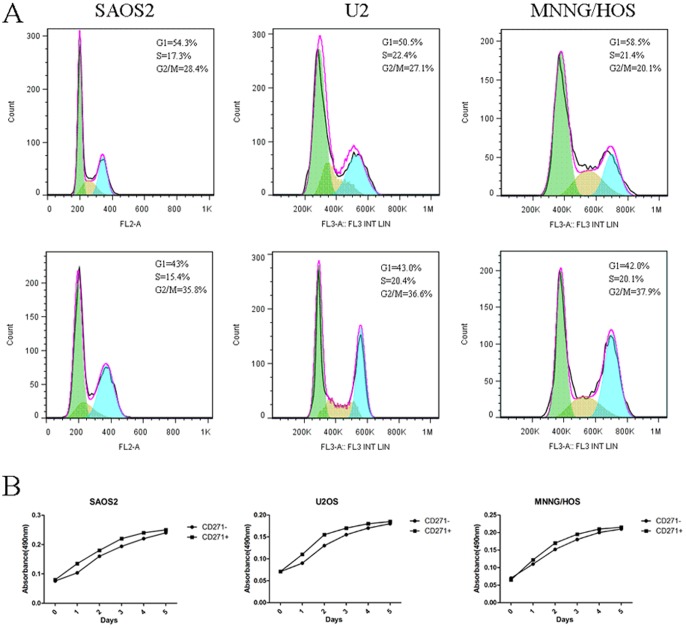
Cell cycle and proliferation analyses. (**A**) In SAOS2, U2OS, MNNG/HOS cell lines, CD271^+^ cells exhibited a higher G2/M peak compared with CD271^−^ cells. (**B**) The growth curves of CD271^+^ cells of all three cells lines display higher proliferative potential compared with CD271^−^ cells.

We investigated the proliferation ability of CD271^+^ and CD271^−^ osteosarcoma cells. As shown in Figure 5B, CD271^+^ cells display higher proliferative potential compared with CD271^−^ cells. The mean doubling time of SAOS2, U2OS, MNNG/HOS derived from CD133^−^ cells were 42 h, 50 h and 45 h, while the cells derived form CD271^+^ cells exhibited a mean doubling time of 36 h, 40 h and 33 h respectively.

In addition, the cell cycle of CD271^+^ cells and CD271^−^ cells was studied. Compared with CD271^−^ cells, CD271^+^ cells exhibited a higher G2/M peak(Figure 5A). The percentages of CD271^+^ cells in G2/M were 35.8%, 36.6%, 37.9% in SAOS2, U2OS, MNNG/HOS respectively, whereas the percentages of CD271^−^ cells in G2/M were 28.4%, 27.1%, 20.1% in the three cell lines.

### CD271^+^ cells were more tumorigenic in vivo compared with CD271^−^ cells

**Figure 6:**
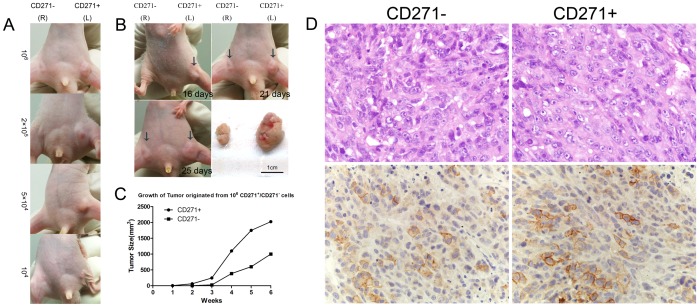
Tumor formation assay in vivo. (**A**)Increasing numbers of CD271^+^ (left) cells were injected subcutaneously in Balb/C-Nu mice with equal amount of CD271^−^ (right) cells as control. (**B**)Gross pictures of tumor originated from 10^6^ CD271^+^/CD271^−^ cells respectively at different time (16, 21, 25days) in one host. (**C**)Growth curve of tumor originated from 10^6^ CD271^+^/CD271^−^ cells showed higher tumorigenic efficiency of CD271^+^ cells compared with CD271^−^ cells.(**D**)Representative images of H&E staining and immunohistochemistry of CD271 expression in tumors originated from CD271^+^ (right panel)and CD271^−^ (left panel)cells. Magnification, 600×.

The most significant characteristic of CSCs is tumorigenicity in vivo. We subcutaneously injected CD271^+^/CD271^−^ cells, isolated by performing MACS, in Balb/C-Nu mice. Only MNNG/HOS were able to generate tumors, rather than SAOS2 and U2OS described as previous research [13]. Up to 6 weeks, the frequency of tumor formation of CD271^+^ cells was higher than that of CD271^−^ cells (Table 1 and Figure 6A). As few as 10^4^ CD271^+^ cells generated tumors while 2×10^5^ CD271^−^ cells were required to form tumors. Furthermore, the size of tumors originated from CD271^+^ cells was considerably greater than that from CD271^−^ cells (Figure 6B and C).

**Table 1:** Tumor formation assay of MNNG/HOS in vivo.

Cell number	1×10^4^	5×10^4^	2×10^5^	1×10^6^
CD271+	1/5	3/5	5/5	4/4
CD271−	0/5	0/5	1/5	1/4

MNNG/HOS osteosarcoma cells were injected subcutaneously into Balb/C-Nu mice. Numbers indicate tumor bearing/injected mice.

Next, immunohistochemisty were performed to investigate the CD271 expression in tumors originated from CD271^+^/CD271^−^ cells respectively. As a result, the tumors originated from CD271^+^/CD271^−^ cells had similar CD271 expression, which was consistent with that of primary cell lines. Apparently, the proportion of CD271^+^ cells was prone to be adjusted to the normal level. This fact also confirmed the ability of differentiation of CD271^+^ cells in vivo.

## Materials and Methods

### Ethics Statement

Ten samples of osteosarcoma patient were retrieved from the Department of Pathology, Qilu Hospital of Shandong University. The specimens were confirmed histopathologically by three pathologists according to the diagnostic criteria. Our research has been approved by the Medical Ethical Committee of Qilu Hospital. A written informed consent document has been obtained from each participant.

Pathogen-free BALB/c nude mice (weighing 17.5–18.9 g, SPF grade, certificate SCXK2011–0012) of 4–5 week old were purchased from Department of Laboratory Animal Science of Peking University (Beijing, China) and maintained at the key Laboratory of Cardiovascular Remodeling and Function Research in Qilu Hospital of Shandong University. The animal experimental protocol complied with the Animal Management Rules of the Chinese Ministry of Health (Document No. 55, 2001) and was approved by Animal Care and Use Committee of Shandong University.

### Cell culture

SAOS2, U2 and MNNG/HOS osteosarcoma cell lines were purchased from the American Type Culture Collection (ATCC). Cells were cultured in McCoy's 5A (Invitrogen, Carlsbad, CA, USA) or DMEM (Invitrogen, Carlsbad, CA, USA) supplemented with 10% FBS (Invitrogen, Carlsbad, CA, USA) at 37°C and 5% CO_2_.

### Sphere formation assay

Cells were seeded at a density of 10^6^/wells in six-well ultralow attachment plates (Corning Inc, Corning, NY). Sphere culture system was constitute of MacCoy's 5A or DMEM, N2 supplement(Sigma Biochemicals, St. Louis, MO), 1% methylcellulose(Sigma Biochemicals, St. Louis, MO), 20 ng/ml EGF(PeproTech, Rocky Hill, NJ) and 20 ng/ml bFGF(PeproTech, Rocky Hill, NJ). Fresh aliquots of EGF and bFGF were added every two days. At 8–10 days, spheres containing at least 50 cells each were observed by inverted phase contrast microscopy (Olympus, Melville, NY).

### Flow cytometry

Cells were detached with trypsin-EDTA into single cell suspension. 10^5^ cells/sample were incubated with AF647-labeled antibodies(Miltenyi Biotec, Italy) or corresponding isotype controls in 4°C for 30 min. After washing steps, the labeled cells were detected using FACScan(BD Calibur) machine. A blank control without labeling was analyzed to delineate the unstained populations.

### Magnetic activated cell sorting

MACS was performed using CD271 MicroBead Kit (Miltenyi Biotec, Italy). Cells were harvested and single-cell suspension was prepared in MACS separation buffer(Miltenyi Biotec, Italy). Cells were incubated with FcR Blocking Reagent and CD271 antibody conjugated to PE in 4°C for 10 min, then incubated with FcR Blocking Reagent and Anti-PE MicroBeads in 4°C for 15 min. After washing steps, magnetic separation was performed using MS Columns and MACS Separator. Flow cytometry was performed to test the purity of sorted cells.

### Western blot

Cells were lysed in RIPA with 1 mM PMSF for 30 min on ice. The mixture was centrifuged at 14,000 g for 5 min and the precipitate was discarded. BCA protein assay (Beyotime, China) was performed to measure the protein concentration. Samples containing equal amount of protein were separated by SDS-PAGE and electroblotted onto polyvinylidene fluoride (PVDF) membrane using standard procedure. Non-specific sites were blocked with 5% nonfat milk for 1 hour at room temperature. PVDF membrane was incubated at 4°C overnight with corresponding primary antibodies, Nanog(Cell Signaling Technology, Beverly, MA, USA), Oct3/4(Cell Signaling Technology, Beverly, MA, USA), STAT3(Cell Signaling Technology, Beverly, MA, USA), ABCG2(Cell Signaling Technology, Beverly, MA, USA), BCL-2(Cell Signaling Technology, Beverly, MA, USA, Beverly, MA, USA),and DNA-PKcs(Abcam, Cambridge, UK). After washed in TBS with 0.1% Tween 20, blots were incubated with anti-rabbit or anti-mouse secondary antibodies conjugated with horseradish peroxidase. Immunoreactive bands were detected by enhanced chemiluminescence(ECL)(GE Healthcare, UK).

### Immunohistochemistry/immunocytochemistry

Tissues of 10 patients who diagnosed as osteosarcoma including 8 osteoblastic osteosarcoma, 5 chondroblastic osteosarcoma, 7 fibroblastic osteosarcoma from Qilu hospital were studied. The paraffin embedded tissue samples were prepared as routine and 3 um sections were mounted on slides. The slides were deparaffinized, rehydrated, and processed for antigen retrieval with citrate antigen retrieval buffer (Beijing Zhongshan Goldenbridge Biotechnology, China). For immunocytochemistry, cells were seeded on slices at confluence of 80–90%. After adherence, cells were fixed with 4% paraformaldehyde. Then both tissue and cell slices were processed as followed.

Endogenous peroxydase was blocked with 3% H_2_O_2_, then slices were incubated with CD271 mouse anti-human primary antibody(Abcam, Cambridge, UK) in 4°C overnight. Signal detection was performed using SP-9000 Histostain-Pluskit(Beijing Zhongshan Goldenbridge Biotechnology, China). After color visualization using ZLI-9031 DAB kit (Beijing Zhongshan Goldenbridge Biotechnology, China), slices were observed under microscope. The CD271 expression was graded according to the method by Manoharan etal [23].

### Drug resistance assessment

Cells were plated in 96-well plate at density of 2000–5000/well. After adherence, different concentrations (0–20 ug/ml) of DDP (Qilu Pharmaceutical Co. Ltd, Shandong, China) were added into the medium for 24 hours and then cells were cultured in drug-free medium for another 24 hours. Cell viability was assessed by MTT assay to determine the IC50 value.

### Cell cycle analysis

CD271^+^ cells and CD271^−^ cells sorted with MACS were washed twice in phosphate-buffered saline and then fixed in 70% ethanol for 60 min at −20°C. After washing in cold phosphate-buffered saline, cells were incubated in 1 mg/ml RNase A for 15 min. Then cells were resuspended in 1 ml of phosphate-buffered saline solution with 40 µg of propidium iodide for 30 min. Samples were then analyzed for their DNA content by FACScan(BD Calibur and Becton Dickinson,CA,USA).

### Tumor formation in vivo

Cells were sorted by MACS and counted by trypan blue staining. Then cells were suspended in non-serum medium with equal volume of Matrigel (BD Biosciences) and injected subcutaneously of Balb/C-Nu mice. Mice were monitored every 2 days for subcutaneous tumors until up to 6 weeks. Tumor volume was calculated using the formula width^2^×length/2.

### Statistical analyses

Statistical analysis was performed using Student's t-test with GraphPad StatMate softeware(GraphPad Softeware, Inc. SanDiego, CA). Values were expressed as mean±SD and level of significance statistically was set at p value <0.05.

## Discussion

In the last two decades, CSC theory has been proposed and studied in many types of neoplasms. Despite the debates of the tumor model [24], compelling evidence has confirmed that a small fraction of cells in tumor display the abilities of self-renewal, differentiation, multi-potency, tumorigenicity, and drug resistance compared with the rest. These cell subpopulations, known as cancer stem cells (CSCs) or tumor-initiating cells (TICs), have been considered to be primarily responsible for the amplification, metastasis, drug resistance and relapse of tumor. Since CSCs are at the apex hierarchy of the heterogeneous cancer cells, identification of CSCs may provide effective approaches for targeted-therapy.

One of the effective methods to isolate CSCs relies on phenotypical analysis. Since first evidence of CSCs was found in human acute myeloid leukemia by Bonnet ect [6], diverse surface markers have been employed to identify CSCs in many types of cancers such as breast tumor, brain tumor, prostate tumor and melanoma [7], [8], [9], [10]. CSCs of osteosarcoma were first characterized by Gibbs [12]. Possibly due to their mesenchymal origin [25], osteosarcoma CSCs are proved to preferentially express MSC markers, such as CD133 [13], [20], CD117/Stro-1 [14], CBX3/ABCA5 [15]. Although no evidence revealed the exact role of CSCs markers in maintaining stemness properties, the certain cellular surface marker phenotype was resulted from the combination effect of microenvironments, genetic and epigenetic changes, as well as intrinsic cellular properties of CSCs. Furthermore, it is believed that more than one type of CSC exists in the tumor mass [11]. Therefore, multiple markers are necessary to identify CSCs.

CD271, known as the neural crest nerve growth factor receptor, is also the surface marker of MCS [16], [17]. In addition, CD271 was reported being expressed in human melanoma-initiating cells [18], which display their distinguished tumorigenicity ability in vivo. In this study, we identified CD271 as the surface marker of osteosarcoma CSCs. We found that CD271^+^ osteosarcoma cells had the abilities of self-renewal, differentiation, drug resistance and tumorigenicity compared with CD271^−^ cells.

CSCs generally account for small proportion of tumor cells. We examined the CD271 expression in human osteosarcoma biopsy and stabilized cell lines and found that a small portion of cells expressed CD271, but the expression was varied markedly among different types of human specimens. Nevertheless, these cells represent a new cell subset by phenotypical feature. Sarcospheres cultured in anchorage-independent, serum-free conditions were widely believed to have stem-like properties and to be rich in CSCs [12]. In our study, CD271^+^ cells had higher proportion in sphere cells than in monolayer cells. This fact strongly indicates CD271^+^ cells have stem-like properties.

Then we investigated the abilities of self-renewal and differentiation of CD271^+^ cells. Sphere formation assay showed that CD271^+^ cells formed more and bigger spheres than CD271^−^ cells. Singly dissociated primary sphere cells could form secondary spheres. Furthermore, in the normal culture condition, the high expression of CD271 was reduced to normal level of primary cell lines. The fact that CD271^+^ cells can rebuild the tumor heterogeneity suggested the capability of differentiation of this cell subpopulation.

In order to study the mechanism of these properties, we investigated the expression of ES cell key marker gene in CD271^+^ cells. Nanog and Oct3/4 are involved in maintaining the self-renewal and pluripotency of undifferentiated ES cells [21]. STAT3 is associated with regulation of cell growth, differentiation and death [26]. CD271^+^ cells had higher expression of these genes maintaining undifferentiation and self-renewal ability of ES cells, which suggested that CD271^+^ cells might have stem-like properties. However, the relationship between these genes and stem-like properties is not fully understood in CSCs. Further research is required to explore the mechanism.

Another characteristic of CSCs was drug resistance, which is thought to be the cause of chemotherapy failure and tumor relapse. In this study, we examined the effect of DDP, a well known anti-tumor agent, on the three osteosarcoma cell lines. The cytotoxic effect of DDP is mainly caused by the formation of cisplatin-DNA adduct which subsequently leads to transcriptional obstacles and double-strand breaks (DSBs). The excess DNA damage ultimately triggers cell death pathway [27]. The catalytic subunit of DNA-dependent protein (DNA-PKcs) plays an important role in non-homologous end joining (NHEJ), which is the major way of DSBs repair [28]. Overexpression of DNA-PKcs gives rise to high survival rate of osteosarcoma cell lines after DDP treatment. Bcl-2, a critical regulator of anti-apoptosis members, promotes cell survival and impairs apoptosis [29]. ATP-binding cassette (ABC) transporters such as ABCB1 and ABCG2, which can efflux hoechst 33342, are expressed in side population(SP) cells enriched in CSCs [30]. In our study, CD271^+^ cells manifested higher expression of DNA-PKcs, Bcl-2 and ABCG2 at protein level. These results suggest that CD271^+^ cells are more refractory to DDP probably due to multiple mechanisms involved in DNA repair, anti-apoptosis and defluxion.

The most important criterion of CSCs is widely believed to be tumorigenicity in vivo. We subcutaneously injected MNNG/HOS cells in immunodeficiency mice with different cell numbers. As shown in our study, CD271^+^ cells had higher frequency of tumor formation. Tumors originated from CD271^+^ cells grew faster than that from CD271^−^ cells. This result supports that CD271^+^ cells are more oncogenic compared with their negative counterparts. Both CD271^+^ and CD271− cells formed tumors with similar expression of CD271 ultimately, which suggested the differentiation ability of CD271^+^ cells.

In this study, we proved that CD271^+^ osteosarcoma cells displayed stem-like properties such as self-renewal, differentiation, drug resistance and tumorigenicity compared with CD271^−^ cells. Low-toxic, targeted therapy based on the research of CSCs surface markers will probably have advantage in the future, compared with traditional cancer treatment including chemotherapy and radiotherapy.

## References

[1] Wu PK, Chen WM, Chen CF, Lee OK, Haung CK, et al (2009) Primary osteogenic sarcoma with pulmonary metastasis: clinical results and prognostic factors in 91 patients. Jpn J Clin Oncol 39: 514–522. 19525290 10.1093/jjco/hyp057

[2] Meyers PA (2009) Muramyl tripeptide (mifamurtide) for the treatment of osteosarcoma. Expert Rev Anticancer Ther 9: 1035–1049. 19671023 10.1586/era.09.69

[3] Meyers PA, Schwartz CL, Krailo M, Kleinerman ES, Betcher D, et al (2005) Osteosarcoma: a randomized, prospective trial of the addition of ifosfamide and/or muramyl tripeptide to cisplatin, doxorubicin, and high-dose methotrexate. J Clin Oncol 23: 2004–2011. 15774791 10.1200/JCO.2005.06.031

[4] Clarke MF (2005) Self-renewal and solid-tumor stem cells. Biol Blood Marrow Transplant 11: 14–16. 15682169 10.1016/j.bbmt.2004.11.011

[5] La Porta CA (2012) Thoughts about cancer stem cells in solid tumors. World J Stem Cells 4: 17–20. 22577494 10.4252/wjsc.v4.i3.17 PMC3348958

[6] Bonnet D, Dick JE (1997) Human acute myeloid leukemia is organized as a hierarchy that originates from a primitive hematopoietic cell. Nat Med 3: 730–737. 9212098 10.1038/nm0797-730

[7] Al-Hajj M, Wicha MS, Benito-Hernandez A, Morrison SJ, Clarke MF (2003) Prospective identification of tumorigenic breast cancer cells. Proc Natl Acad Sci U S A 100: 3983–3988. 12629218 10.1073/pnas.0530291100 PMC153034

[8] Singh SK, Clarke ID, Terasaki M, Bonn VE, Hawkins C, et al (2003) Identification of a cancer stem cell in human brain tumors. Cancer Res 63: 5821–5828. 14522905

[9] Tang DG, Patrawala L, Calhoun T, Bhatia B, Choy G, et al (2007) Prostate cancer stem/progenitor cells: identification, characterization, and implications. Mol Carcinog 46: 1–14. 16921491 10.1002/mc.20255

[10] Schatton T, Murphy GF, Frank NY, Yamaura K, Waaga-Gasser AM, et al (2008) Identification of cells initiating human melanomas. Nature 451: 345–349. 18202660 10.1038/nature06489 PMC3660705

[11] Basu-Roy U, Basilico C, Mansukhani A (2013) Perspectives on cancer stem cells in osteosarcoma. Cancer Lett 338: 158–167. 22659734 10.1016/j.canlet.2012.05.028 PMC3552024

[12] Gibbs CP, Kukekov VG, Reith JD, Tchigrinova O, Suslov ON, et al (2005) Stem-like cells in bone sarcomas: implications for tumorigenesis. Neoplasia 7: 967–976. 16331882 10.1593/neo.05394 PMC1502023

[13] Tirino V, Desiderio V, d'Aquino R, De Francesco F, Pirozzi G, et al (2008) Detection and characterization of CD133+ cancer stem cells in human solid tumours. PLoS One 3: e3469. 18941626 10.1371/journal.pone.0003469 PMC2565108

[14] Adhikari AS, Agarwal N, Wood BM, Porretta C, Ruiz B, et al (2010) CD117 and Stro-1 identify osteosarcoma tumor-initiating cells associated with metastasis and drug resistance. Cancer Res 70: 4602–4612. 20460510 10.1158/0008-5472.CAN-09-3463 PMC3139225

[15] Saini V, Hose CD, Monks A, Nagashima K, Han B, et al (2012) Identification of CBX3 and ABCA5 as putative biomarkers for tumor stem cells in osteosarcoma. PLoS One 7: e41401. 22870217 10.1371/journal.pone.0041401 PMC3411700

[16] Pontikoglou C, Deschaseaux F, Sensebe L, Papadaki HA (2011) Bone marrow mesenchymal stem cells: biological properties and their role in hematopoiesis and hematopoietic stem cell transplantation. Stem Cell Rev 7: 569–589. 21249477 10.1007/s12015-011-9228-8

[17] Rasini V, Dominici M, Kluba T, Siegel G, Lusenti G, et al (2013) Mesenchymal stromal/stem cells markers in the human bone marrow. Cytotherapy 15: 292–306. 23312449 10.1016/j.jcyt.2012.11.009

[18] Boiko AD, Razorenova OV, van de Rijn M, Swetter SM, Johnson DL, et al (2010) Human melanoma-initiating cells express neural crest nerve growth factor receptor CD271. Nature 466: 133–137. 20596026 10.1038/nature09161 PMC2898751

[19] Fujii H, Honoki K, Tsujiuchi T, Kido A, Yoshitani K, et al (2009) Sphere-forming stem-like cell populations with drug resistance in human sarcoma cell lines. Int J Oncol 34: 1381–1386. 19360350

[20] Tirino V, Desiderio V, Paino F, De Rosa A, Papaccio F, et al (2011) Human primary bone sarcomas contain CD133+ cancer stem cells displaying high tumorigenicity in vivo. FASEB J 25: 2022–2030. 21385990 10.1096/fj.10-179036

[21] Chambers I, Colby D, Robertson M, Nichols J, Lee S, et al (2003) Functional expression cloning of Nanog, a pluripotency sustaining factor in embryonic stem cells. Cell 113: 643–655. 12787505 10.1016/s0092-8674(03)00392-1

[22] Mitsui K, Tokuzawa Y, Itoh H, Segawa K, Murakami M, et al (2003) The homeoprotein Nanog is required for maintenance of pluripotency in mouse epiblast and ES cells. Cell 113: 631–642. 12787504 10.1016/s0092-8674(03)00393-3

[23] Manoharan A, Horsley R, Pitney WR (1979) The reticulin content of bone marrow in acute leukaemia in adults. Br J Haematol 43: 185–190. 508627 10.1111/j.1365-2141.1979.tb03740.x

[24] Greaves M, Maley CC (2012) Clonal evolution in cancer. Nature 481: 306–313. 22258609 10.1038/nature10762 PMC3367003

[25] Mohseny AB, Szuhai K, Romeo S, Buddingh EP, Briaire-de Bruijn I, et al (2009) Osteosarcoma originates from mesenchymal stem cells in consequence of aneuploidization and genomic loss of Cdkn2. J Pathol 219: 294–305. 19718709 10.1002/path.2603

[26] Wang BX, Platanias LC, Fish EN (2013) STAT activation in malignancies: roles in tumor progression and in the generation of antineoplastic effects of IFNs. J Interferon Cytokine Res 33: 181–188. 23570384 10.1089/jir.2012.0154

[27] Gonzalez VM, Fuertes MA, Alonso C, Perez JM (2001) Is cisplatin-induced cell death always produced by apoptosis? Mol Pharmacol 59: 657–663. 11259608 10.1124/mol.59.4.657

[28] Lieber MR (2010) NHEJ and its backup pathways in chromosomal translocations. Nat Struct Mol Biol 17: 393–395. 20368722 10.1038/nsmb0410-393 PMC3074614

[29] Cory S, Adams JM (2002) The Bcl2 family: regulators of the cellular life-or-death switch. Nat Rev Cancer 2: 647–656. 12209154 10.1038/nrc883

[30] Hadnagy A, Gaboury L, Beaulieu R, Balicki D (2006) SP analysis may be used to identify cancer stem cell populations. Exp Cell Res 312: 3701–3710. 17046749 10.1016/j.yexcr.2006.08.030

